# Neuropathologic investigation of phospho‐threonine 217 provides neurobiologic insights into the intersection between amyloid‐β and tau

**DOI:** 10.1002/alz.089414

**Published:** 2025-01-03

**Authors:** Melissa E. Murray

**Affiliations:** ^1^ Mayo Clinic, Jacksonville, FL USA

## Abstract

**Background:**

Posttranslational modifications of tau occurs throughout disease progression in Alzheimer’s disease (AD) and non‐AD tauopathies. Phosphorylation of tau (p‐tau) in the proline‐rich region is a common target of immunohistochemical and fluid biomarker evaluation. P‐tau217 has emerged as a highly accurate fluid biomarker in AD, however elevated levels are not observed in non‐AD tauopathies.

**Method:**

A neuropathologic overview of tau lesions immunopositive for phosphorylation at threonine 217 (pT217) in AD and non‐AD tauopathies will be provided in a didactic format with an emphasis on disease spectrum. The relevant biomarker literature with neuropathologic validation will be highlighted.

**Result:**

Immunohistochemical evaluation of pT217 in AD demonstrates immunostaining in pretangles and mature tangles, but rarely observed in ghost tangles. Neuritic pathology in AD with pT217 immunopositivity include both neuropil threads and dystrophic neurites that cluster in neuritic plaques. Lesions in non‐AD tauopathies are also immunopositive for pT217, as exampled by immunolabelling of argyrophilic grains and tufted astrocytes. Digital pathology measures of pT217 and amyloid‐β will be compared with plasma p‐tau217 levels to contextualize the relationship. Additionally, the relationship between neurotransmitter hub vulnerability will be explored to consider upstream factors that may influence soluble tau release into plasma.

**Conclusion:**

Neuropathologic insights discussed through visual and quantitative comparison of insoluble phospho‐tau accumulation and soluble levels of p‐tau217 may provide neurobiologic insights into the intersection between amyloid‐β and tau in the human brain.

